# Position Statement on the Diagnosis, Treatment, and Response Evaluation to Systemic Therapies of Advanced Neuroendocrine Tumors, With a Special Focus on Radioligand Therapy

**DOI:** 10.1093/oncolo/oyab041

**Published:** 2022-03-07

**Authors:** Jaume Capdevila, Enrique Grande, Rocío García-Carbonero, Marc Simó, Mª Isabel del Olmo-García, Paula Jiménez-Fonseca, Alberto Carmona-Bayonas, Virginia Pubul

**Affiliations:** 1 Department of Medical Oncology, Vall Hebron University Hospital, Vall Hebron Institute of Oncology (VHIO), IOB-Quiron-Teknon Barcelona, Barcelona, Spain; 2 Department of Medical Oncology, MD Anderson Cancer Center, Madrid, Spain; 3 Department of Medical Oncology, Hospital Universitario Doce de Octubre, Madrid, Spain; 4 Department of Nuclear Medicine and Molecular Imaging, Hospital Universitari Vall d’Hebron, Barcelona, Spain; 5 Department of Endocrinology, Hospital Universitario y Politécnico la Fe de Valencia, Valencia, Spain; 6 Department of Medical Oncology, Hospital Universitario Central de Asturias, ISPA, Madrid, Spain; 7 Department of Hematology and Medical Oncology, Hospital General Universitario Morales Meseguer, University of Murcia, IMIB, CP13/00126, PI17/0050 (ISCIII & FEDER) and Fundación Séneca (04515/GERM/06), Murcia, Spain; 8 Department of Nuclear Medicine Department and Molecular Imaging Research Group, University Hospital and Health Research Institute of Santiago de Compostela (IDIS), Santiago de Compostela, Spain

**Keywords:** neuroendocrine tumors, advanced, peptide receptor radionuclide therapy, progression, sequencing, neoadjuvant therapy, retreatment

## Abstract

**Background:**

The aim of this study was to provide a guidance for the management of neuroendocrine tumors (NETs) in clinical practice.

**Material and Methods:**

Nominal group and Delphi techniques were used. A steering committee of 8 experts reviewed the current management of NETs, identified controversies and gaps, critically analyzed the available evidence, and formulated several guiding statements for clinicians. Subsequently, a panel of 26 experts, was selected to test agreement with the statements through 2 Delphi rounds. Items were scored on a 4-point Likert scale from 1 = totally agree to 4 = totally disagree. The agreement was considered if ≥75% of answers pertained to Categories 1 and 2 (consensus with the agreement) or Categories 3 and 4 (consensus with the disagreement).

**Results:**

Overall, 132 statements were proposed, which incorporated the following areas: (1) overarching principles; (2) progression and treatment response criteria; (3) advanced gastro-enteric NETs; (4) advanced pancreatic NETs; (5) advanced NETs in other locations; (6) re-treatment with radioligand therapy (RLT); (7) neoadjuvant therapy. After 2 Delphi rounds, only 4 statements lacked a clear consensus. RLT was not only recommended in the sequencing of different NETs but also as neoadjuvant treatment, while several indications for retreatment with RLT were also established.

**Conclusion:**

This document sought to pull together the experts’ attitudes when dealing with different clinical scenarios of patients suffering from NETs, with RLT having a specific role where evidence-based data are limited.

**Implications for Practice**

During the past years, significant advances in NETs’ molecular biology, diagnostic techniques, and therapeutic options/strategies have emerged, especially for patients with advanced or metastatic disease. The experts agree on that clinical symptoms, biological markers, morphological/radiological imaging l, or functional/nuclear medicine imaging alone, do not determine disease progression or treatment response. Regarding NET treatment, RLT has a relevant role in the treatment of different types of NETs, as well as neoadjuvant therapy, in the retreatment, and even to achieve resectability in certain patients with metastases.

## Introduction

Neuroendocrine tumors (NETs) are a heterogeneous and complex group of neoplasms that arise from neuroendocrine cells and mainly affect gastro-entero-pancreatic (GEP) tissues, as well as other locations such as the lung or thymus.^1^ Over the last decades, the incidence of NETs has increased up to around 3.65 per 100 000 individuals per year, and they particularly affect the elderly.^1-3^ These tumors are generally diagnosed within the fifth decade of life, with men being affected slightly more often than women; around 5% of these growths are associated with hereditary predisposition syndromes.^1-3^ There is a wide spectrum of disease, ranging from slow-growing, indolent, and incidentally detected tumors to highly aggressive malignancies with poor prognosis. However, at diagnosis, around 40%-50% of patients living with NET already present with distant metastases.^4^ More specifically, at diagnosis, approximately 65%-95% of GEP NETs (excluding appendiceal, gastric, and rectal NETs) are already associated with hepatic metastases.^1,5^ This is paramount, as hepatic metastases represent the most critical prognostic factor.^6^

During the past several years, significant advances in NETs molecular biology, diagnostic techniques, and therapeutic options/strategies have emerged, for the most part, concerning patients with advanced or metastatic NETs, resulting in earlier diagnosis while improving both prognosis and quality of life.^7,8^ Currently, the 5-year survival rate is estimated at up to 80%-85% in patients with well-differentiated NETs and up to 13%-54% in patients with NETs and hepatic metastases.^5,9^

Surgery remains the only potentially curative therapeutic strategy in patients with NETs.^10^ On the other hand, medical treatment for these patients consists of long-acting somatostatin analogs (SSA) including octreotide and lanreotide, chemotherapy, targeted therapies like everolimus and sunitinib, loco-regional therapies, as well as radioligand therapy (RLT).^11^ The proper therapy selection depends on different factors, with some of them related to the patient (age, global health, etc.), and others to the tumor including its proliferative activity, somatostatin receptor (SSTR) expression, tumor growth rate, or disease extension.

Consensus/position documents and clinical guidelines on NETs management seek to analyze the best available evidence to provide some guidance for treatment decision-making, especially concerning sequencing therapies in situations where evidence is insufficient or even totally absent.^11-22^ These documents usually cover the most relevant patient and tumor phenotypes. However, in daily practice, there are often complex “borderline” scenarios or clinical situations that are not specifically covered by these documents. Consequently, physicians may face different types of patients with NET in their daily practice that prove to be really challenging.

Therefore, this consensus document sought to provide a guide for managing patients with NET, while focusing on those areas that likely generate clinical questions or controversies, such as the attitude to adopt in patients living with advanced diseases, with RLT’s role being specially highlighted.

## Material and Methods

### Study Design

A qualitative study was performed. Nominal group and Delphi techniques were applied to elaborate the consensus, with a comprehensive narrative review supporting the statements. An external expert in Delphi methodology guaranteed the quality of the overall process. This study was conducted in accordance with Good Clinical Practice and the current version of the revised Declaration of Helsinki (World Medical Association Declaration of Helsinki).

### Expert Panel Selection and Clinical Statement Generation

In a first step, a multidisciplinary steering committee of 8 experts on NETs was established. They were responsible for: (1) selection of the expert panel; (2) identification of current relevant clinical questions/controversies in the management of advanced NETs; (3) generation of clinical statements to answer these questions/controversies based on their experience and an extensive and critical narrative literature review. These statements were organized in 7 main sections including: (a) overarching principles; (b) progression and treatment response criteria; (c) advanced GEP NETs; (d) advanced pancreatic NETs; (e) advanced NETs in other locations; (f) re-treatment using RLT; (g) neoadjuvant therapy; (4) definition of the consensus levels and agreement on methodology; (5) interpretation of the results from the Delphi rounds; (6) final edition of the consensus document. The steering committee did not participate in the Delphi rounds.

The expert panel comprised 26 experts that were selected according to the following criteria: (1) medical oncologist; (2) specialized in NETs with proven clinical experience of ≥8 years or ≥5 publications; (3) member of the Spanish Society of Medical Oncology (SEOM) or Spanish Task Force for Neuroendocrine and Endocrine Tumors (GETNE). In the selection process, a balanced territorial representation of Spain was considered.

### Delphi Process

The expert panel completed 2 Delphi rounds through an online platform. After each round, a facilitator provided an anonymous summary of the experts’ forecasts from the previous round, as well as the reasons they put forth for their judgments. In the first round, the panelists voted using a 4-point Likert scale from 1 = totally agree to 4 = totally disagree. The agreement was considered if ≥ 75% of answers pertained to Categories 1 and 2 (consensus with the agreement) or Categories 3 and 4 (consensus with the disagreement). All statements reaching consensus in the first round did not undergo the second Delphi round. The remaining statements were analyzed by the steering committee to discern whether the lack of consensus was due to the ambiguity of the statement itself, in which case it was reformulated, or due to the issue being controversial itself, in which case the statement remained unchanged for the second Delphi round. In the second Delphi round, the statements were voted using the same categories as described for the first round. At this stage, the same criteria were established as in the first round for “consensus” in agreement and disagreement. When the response rates in Categories 1 and 2 or Categories 3 and 4 were 60%-70%, it was considered “majority”, and when it was <60%, it was called “dissent”. Finally, if this rate reached 100%, it was considered “unanimous”.

### Edition of the Document

The steering committee wrote the position statement document. A comprehensive literature search in MEDLINE was performed to support the statements.

## Results

### Delphi Results

Overall, 132 statements were generated (Supplementary Tables S1-S7 depict Delphi results). In the first Delphi round, 6 statements reached the required level of agreement and did thus not pass to the second round, while 10 were reformulated. After the second round, the agreement level with the 132 statements was as follows: unanimous 26 (5 with agreement; 21 with disagreement), 78 consensus (41 with agreement; 37 with disagreement), 24 majorities (9 with agreement; 15 with disagreement), and 4 dissents. Another statement of the RLT retreatment section was finally rejected, as it was probably misunderstood (Statement 66 of the supplementary material).

### Overarching Principles

There has been a broad consensus among experts (>80%) to consider that: (1) Patients with a recent NETs diagnosis should be discussed within a multidisciplinary NETs committee; (2) Implementation of improved nuclear medicine diagnostic techniques (especially ^68^Ga-PET-CT) has a relevant impact on treatment decision-making in patients living with NET; (3) ^177^Lu-DOTATATE has restructured treatment schemes for patients with gastrointestinal and pancreatic NET (Supplementary Table S1).

### Progression and Response to Treatment Criteria

Disease progression and treatment response evaluation are key areas in advanced NET monitoring. However, in recent years, different factors have led to reformulating/defining disease progression and treatment response criteria, including NET heterogeneity, knowledge advances, improved imaging techniques, or new treatments and strategies.^23-25^

The experts agree (Supplementary Table S2) clinical symptoms, biological markers like chromogranin A (CgA), morphological/radiological imaging like RECIST v 1.1, or functional/nuclear medicine imaging do not determine disease progression or treatment response. All these display several limitations that must be considered.

A recurrence or worsening of functional symptoms due to hormone production or new symptom occurrences may suggest disease progression. However, their prior absence, as well as their reproducibility, is uncertain.^26,27^ In recent years, there has been an increased interest in quality of life as a surrogate marker of disease progression, but the respective results are still preliminary.^28^

CgA displays low sensitivity, with high within- and between-subject variations, thus causing many false positives.^29,30^ On the other hand, after excluding conditions with elevated gastrin levels, CgA levels have been shown to correlate with tumor burden. Moreover, recent clinical trials have demonstrated its prognostic value.^31-34^

A significant increase in the standardized uptake value (SUV) in positron emission tomography (PET) was not considered sufficient to ascertain disease progression. Progression is not only determined by an increase in tumor activity but also by the development of new lesions.^24,35^

Concerning RECIST v 1.1, although these criteria are still widely used for establishing disease progression and treatment response, their usefulness has been questioned for different reasons. Several studies have shown that tumor response to targeted therapies is rarely associated with shrinkage, as opposed to prolonged progression-free survival (PFS). Thus, other criteria like those of CHOI may be more useful for patients on targeted therapies.^26,36-39^ Furthermore, discordances have been observed between longer PFS values and low RECIST-assessed response rates (<10%),^40^ in addition to difficulties in assessing liver metastases, given that RECIST thresholds are not suited for the slow evolution of many NETs, which may thus be misclassified as SD.^24,41^

There was dissent about the consideration that CHOI criteria only apply to patients on anti-angiogenic targeted therapies.^36^ Preliminary data from patients with advanced NET suggest that CHOI criteria could help identify patients who might already benefit from targeted therapy at an earlier time point.^37,38^ Therefore, although there is not yet any robust evidence on this issue, CHOI criteria could potentially be used to assess the response to other treatment types.

### Sequencing of Treatments in Gastrointestinal NETs

In this section, we discuss uncertainties that usually arise in clinical practice upon selecting the most appropriate treatment and sequencing of therapies in patients with gastrointestinal NET, especially when different therapeutic options are possible, yet without any head-to-head clinical comparative trials available (see Table 1).

**Table 1.** Treatments for patients with advanced gastrointestinal neuroendocrine tumors.

Tumor characteristics				Most appropriated treatment
Advanced small intestinal NETs	G2	Peritoneal carcinomatosis	Progression to SSAs	- RLT
Advanced gastrointestinal NETs	G2	Multiple bone metastases	Progression to SSAs	- RLT
	G1-G2	Functional with uncontrolled hormonal symptoms and hepatic metastases	Progression to SSAs	- RLT - Loco-regional therapy

Abbreviations: NETs, neuroendocrine tumors; G, grade; SSAs, somatostatin analogs; RLT, radioligand therapy.

First, in patients with G1-G2 small intestine NET, the panel agrees (89% consensus) that both the efficacy and safety of RLT are superior to those displayed by everolimus, whereas this is not the case in G1-G2 NETs of other intestinal locations. The NETTER-1, a phase III randomized controlled trial (RCT) involving 229 patients with advanced small bowel NETs, revealed an estimated 20-month PFS with ^177^Lu-DOTATATE of 65.2% versus 10.8% with octreotide, with a response rate of 18% versus 3% (*P* < .001), respectively, along with an acceptable safety profile.^42,43^ Data of colorectal NETs treated with ^177^Lu-DOATATE are currently scarce. Several studies have analyzed the everolimus activity in different NET subtypes. However, the number of included patients with small bowel NETs was inferior to that of patients included with other NET subtypes. In the RADIANT-2 phase III RCT, the patient subgroup with advanced small bowel NETs displayed a median PFS of 18.6 months with everolimus plus octeotride long-acting release (LAR) versus 14 months with octeotride LAR alone. In another RADIANT-2 subanalysis, patients with advanced colorectal NETs displayed a median PFS of 29.9 months with everolimus plus octeotride LAR versus 6.6 months with octeotroide LAR alone.^44^ Finally, in the RADIANT-4 phase III RCT, a placebo-controlled trial, PFS was superior with everolimus in small bowel NETs (hazard ratio [HR] = 0.71; 95% CI, 0.40-1.26) and in NETs of other locations (HR = 0.27; 95% CI, 0.15-0.51).^45^

In patients with small intestine G2 NETs, peritoneal carcinomatosis-only metastases, and in those displaying progression under SSA, majority of the Delphi participants (74%) considered RLT the most appropriate option as compared to others, such as everolimus or temozolamide plus capecitabine (TEMCAP). Chemotherapy has proven very effective in these cases.^19^ Despite the lack of robust data regarding everolimus or RLT, the NETTER-1 study included some patients suffering from small bowel NETs with peritoneal carcinomatosis.^42^

There was consensus (85%) in using RLT as the most suitable therapy in gastrointestinal G2 NETs with multiple bone metastases and in progression under SSAs, in comparison with other options like everolimus or TEMCAP. Chemotherapy in NETs with a high bone tumor burden may cause significant hematological toxicity.^20^ On the other hand, as data are not available for everolimus,^46,47^ clinical guidelines consider RLT a valid and promising option,^20^ although the available data are still preliminary.

Among therapeutic options for patients living with G1-G2 functional gastrointestinal NET with liver metastases, who are progressing under SSA with uncontrolled hormonal symptoms, RLT (96% consensus) and loco-regional treatments (majority, 73%) were considered the most appropriate modalities. A subanalysis of the NETTER-1 study demonstrated a statistical and clinically significant increase in PFS with ^177^Lu-DOTATATE in patients exhibiting low/moderate/high liver tumor burden.^48^ Within the ^177^Lu-DOTATATE arm, no significant difference in PFS was observed between patients with low, moderate, or high baseline tumor burden (*P* = .722). However, in the high-dose octreotide arm, a significant correlation was found between liver tumor burden and PFS, with median PFS of 9.1, 8.7, and 5.4 months for patients with low, moderate, and high burdens, respectively (*P* = .0169).^48^ Loco-regional treatments in small observational studies have achieved 5-year overall survival (OS) rates of 50%-80% in gastrointestinal NETs.^49,50^ Notably, these treatments are also recommended by most international clinical guidelines.^11,39^

Concerning nonfunctional gastrointestinal NETs progressing under long-acting SSAs, when RLT is prescribed, the Delphi participants agreed (consensus, 81%) to discontinue long-acting SSAs 4 weeks before RLT, and restart SSAs approximately 24 hours after each RLT infusion, and to use them monthly after RLT treatment completion upon progression.^42^ Indeed, somatostatin and its analogs bind competitively to SSTRs and may interfere with RLT efficacy. As stated in the summary of product characteristics (SPC), administration of long-acting SSA should be avoided within 30 days of RLT. However, in functional gastrointestinal NETs progressing under long-acting SSAs with uncontrolled symptoms, if RLT is prescribed, the most accepted option (85%) was to stop long-acting SSAs and initiate short-acting octreotide 48 hours before RLT, in alignment with the product’s SPC.

Most of Delphi participants (88%) disagreed with the following statement: The short/long-term RLT toxicity proves to be lower if it is given to patients that are in progression under SSAs compared to other treatments like everolimus. There was no robust and direct evidence in this regard; although cumulative toxicity must be considered, RLT has demonstrated an acceptable safety profile regardless of prior therapy.^51,52^

### Sequencing of Treatments in Pancreatic NETs

We now describe the main statements for pancreatic NETs (see Table 2).

**Table 2.** Treatments for patients with advanced pancreatic neuroendocrine tumors.

Tumor characteristics				Most appropriated treatment
Advanced G1 pancreatic NETs	SSTR scintigraphy +	Radiologic progression under SSAs >3 years	Asymptomatic and low tumor burden	- Targeted therapy
		Radiographic progression under SSAs <3 years	Functional	- RLT
Advanced G2 pancreatic NETs			Non-functional, symptomatic and high tumor burden	- Chemotherapy (before RLT)

Abbreviations: NETs, neuroendocrine tumors; G, grade; SSAs, somatostatin analogs; RLT, radioligand therapy; SSTR, somatostatin receptor.

As stated in international clinical guidelines^11,15,18^ and supported by the evidence from the CLARINET study,^53^ there has been agreement in recommending (consensus 81%) systemic treatment with SSAs as a single-agent in patients with advanced G1-G2 pancreatic NETs, unless there is a contraindication for this.

Considering patients with advanced pancreatic NETs, the experts identified several clinical and imaging factors that may help to select a particular therapy.

In patients with asymptomatic advanced G1 pancreatic NET, SSTR expression, low tumor burden, and documented radiological progression -after over 3 years of SSAs, second-line treatment with targeted therapy (everolimus and sunitinib) would be the preferred option (consensus, 88%). The RADIANT-3 Phase III RCT, which was specifically conducted in advanced pancreatic NET patients, reported a median PFS of 11 months with everolimus versus 4.6 with placebo (*P* < .001), along with an estimated rate of patients that were alive and progression-free at 18 months of 95% versus 34%, respectively. Most undesirable effects reported with everolimus were of G1 or G2.^54^ The sunitinib efficacy was evaluated in another phase III RCT in advanced pancreatic NET patients.^40^ The median PFS was 11.4 months with sunitinib versus 5.5 with placebo (*P* < .001), and the objective response rate was 9.3% versus 0%, respectively.^40^

In patients with advanced G1 functional pancreatic NET, SSTR expression and documented radiological progression after less than 3 years of SSAs, second-line RLT would be the preferred option for most of the Delphi participants (consensus, 92%). Despite the lack of data from specific Phase III RCTs, there is positive evidence arising from observational studies and Phase I/II trials, whereas the RLT efficacy in functional NETs is already known.^43^ Moreover, recent systematic literature reviews and meta-analyses have demonstrated both RLT’s efficacy and safety in advanced pancreatic NET patients.^55,56^

Considering patients with advanced insulinoma progressing under SSAs or with uncontrolled symptoms, there was a consensus (81%) that everolimus would be preferred over RLT. Data from observational studies have shown that everolimus is an effective treatment for patients with metastatic insulinoma and refractory hypoglycemia.^57-59^ In addition, inhibition of the mTOR pathway was shown to decrease insulin secretion in patients suffering from insulinomas.^57-59^ Preliminary published data suggest that RLT can prove successful in further controlling severe hypoglycemia in malignant insulinomas.^60^

We found that 62% of Delphi participants recommend using at least one SSA, targeted therapy, or chemotherapy (streptozocin-based or temozolomide-based chemotherapy) before considering RLT in G1-G2 pancreatic NETs with SSTR expression. Currently, there is no evidence as to the best treatment sequencing.

In this section, consensus (96%) was reached on considering chemotherapy (streptozocin-based or temozolomide-based chemotherapy) prior to RLT in advanced, nonfunctional G2 pancreatic NET patients suffering from symptoms due to high tumor burden. Available evidence from clinical trials^61-64^ and expert recommendations support the use of chemotherapy in these cases.^11,18,39^

### Treatment Sequencing in Other NETs

This project also discussed the most appropriate treatments for patients with NETs in other locations (see Table 3).

**Table 3.** Treatments for patients with advanced pheochromocytomas/paragangliomas and bronchial neuroendocrine tumors.

Tumor characteristics				Most appropriated treatment
Pheochromocytomas/paragangliomas	SSTR expression and MIBG positive	Progressing	Nonfunctional	MIBG ^177^Lu-DOTATATE
			Functional with uncontrolled hormonal symptoms	MIBG ^177^Lu-DOTATATE SSAs
Bronchial NETs	G1-G2	Positive SSTR imaging	1^st^ line	SSAs
			2^nd^line (progression to SSAs)	Everolimus ^177^Lu-DOTATATE
			Positive FDG-PET-TC	Chemotherapy(temozolomide and capecitabine) Everolimus

Abbreviations: NETs, neuroendocrine tumors; G, grade; SSTR, somatostatin receptor; MIBG, meta-iodobenzylguanidine; FDG-PET-TC, 2-fluoro-2-deoxy-d-glucose-positron-electron tomography-computed tomography; SSAs, somatostatin analogs.

There was a significant consensus (96%) on considering ^177^Lu-DOTATATE for patients with advanced pheochromocytomas/paragangliomas (PPGL) with proven SSTR expression. A systematic literature review including meta-analysis on RLT efficacy and safety in patients with advanced PPGL was retrieved.^65^ This review, which comprised 12 observational studies, reported an objective response rate of 25% and disease control rate of 84%. RLT was associated with the following Grade 3 or 4 undesirable effect rates: neutropenia 3%, thrombocytopenia 9%, lymphopenia 11%, and nephrotoxicity 4%.^65^

Similarly, in progressive advanced nonfunctional PPGL patients, positive metaiodobenzylguanidine (MIBG) and SSTR expression, ^131^I-MIBG (consensus, 92%) and ^177^Lu-DOTATATE (consensus, 77%) were considered the most appropriate treatments. However, in functional tumors with uncontrolled hormonal symptoms, in addition to MIBG (consensus, 92%) and ^177^Lu-DOTATATE (consensus, 85%), SSAs (consensus, 77%) were considered the most suitable treatments. In phase II RCTs of ^131^I-MIBG, the objective response rate (RECIST criteria) was around 25%, while long-term survival reached up to 6 years.^66,67^^131^I-MIBG-emergent undesirable effects of Grade 3-4 were reported in 16%-83% of patients.^66-68^ As described before, there are promising data concerning ^177^Lu-DOTATATE in advanced PPGL.^65^ Concerning SSAs, the evidence in these patients is still scarce.

Advanced PPGL patients with SSTR expression and FDG-PET-CT uptake were analyzed upon disease progression. Among Delphi participants, different levels of disagreement were obtained regarding the most appropriate therapy, including ^177^Lu-DOTATATE, SSAs, or chemotherapy (cyclophosphamide plus vincristine plus dacarbazine or temozolomide).

Concerning G1-G2 advanced bronchial NET patients with SSTR expression, the following strategy was considered appropriate: first-line treatment with SSAs (unanimity, 100%), everolimus (consensus, 96%), and ^177^Lu-DOTATATE (majority, 65%); second-line at progression under SSAs, and if FDG-PET-CT uptake, chemotherapy (TEMCAPE) (consensus, 96%) or everolimus (majority, 73%). The efficacy and safety of first-line SSA treatment in advanced bronchial NETs have been evaluated in observational studies, showing symptom control.^69^ Although there were no data confirming tumor growth control in advanced bronchial NET patients, positive experiences were made in tumors of other locations.^53,70^ A subgroup analysis of RADIANT-4 study involving patients with advanced nonfunctional bronchial NETs, with some of them progressing under SSAs, revealed a significant improvement in PFS with everolimus versus placebo, namely 11 months versus 3.9 months (*P* < .001), with tumor shrinkage seen in a higher percentage of patients (58% vs 13%), and a trend toward longer OS.^71,72^ Similarly, a retrospective observational study demonstrated everolimus efficacy in patients with advanced, progressive, and well-differentiated NETs regardless of FDG-PET-TC uptake.^73^ For ^177^Lu-DOTATATE, evidence for advanced bronchial NET patients was retrieved from small observational studies and a phase III RCT involving 23 patients. Observed overall response rates ranged from 13% to 30%, PFS from 19 to 28 months, and OS from 32 to 59 months.^74-77^ Chemotherapy (TEMCAP) has proven effective in small observational studies involving advanced NET patients.^64,78^ All these considerations very much aligned with clinical guidelines.^13,18^

Considering G1-G2 advanced bronchial NET patients, the following were considered key factors for selecting ^177^Lu-DOTATATE: SUV uptake in Gallium-PET (consensus, 93%), progression under everolimus (consensus, 92%), or progression under SSAs (consensus, 92%). As previously mentioned, in the absence of robust data and considering its efficacy and safety profile, ^177^Lu-DOTATATE would currently be reserved for second- or third-line treatment in advanced bronchial NET patients.^13,79^

### RLT Re-treatment

Concerning RLT re-treatment, the Delphi participants agreed that patients responding to a first RLT course could respond to subsequent RLT courses (consensus, 96%).

The efficacy and safety of RLT re-treatment have been analyzed in small observational studies, in patients with progressing NETs at different sites and stages. There was a great variability in the number of cycles (up to 9 cycles) and cumulative doses administered.^80-89^ The published median PFS with RLT re-treatment varied from 6 to 22 months. There were also case studies retrieved reporting up to 4 RLT re-treatment courses, with median PFS of 18.9, 12.1, 9.3, and 4.3 months, respectively.^87^ Median OS varied depending on the study, ranging from 9 to 93.9 months.^80-89^ Recently, 2 systematic literature reviews and meta-analyses have found (aggregated data) an objective response rate of 17.1%, and disease control rate of 76.9% with RLT re-treatment in advanced NET patients.^90,91^

As maximum cumulative doses of ^177^Lu-DOTATATE have yet not been established, a Delphi agreement was attained that RLT re-treatment may be preferred over targeted therapy in pancreatic NET patients with a long-term response (consensus, 77%). In the literature, many patients on RLT re-treatment responded to this therapy for more than 1 year before experiencing progression.^90,91^ For the panelists, RLT re-treatment is also likely to be an option in well- or moderately differentiated NET patients, especially with a Ki67 of 10%-20% (100% unanimity). Notably, the RLT re-treatment efficacy has been mainly analyzed in well-differentiated NET patients.^90,91^

For RLT re-treatment, the Delphi members agreed on the following selection criteria: time to progression, previous objective response, tumor burden, location of the primary NET, and subsequent treatment options.

Likewise, an agreement was reached to consider RLT re-treatment if the time to progression was 12 months at least (consensus, 96%). Many published studies have additionally established a PFS >18 months from the first RLT cycle as a criterion indicative for RLT re-treatment.^80-88,90,91^

Safety issues were evaluated, as well. Disagreement was obtained with a statement suggesting that there was no increase in the incidence of myelodysplastic syndrome or acute leukemia observed with RLT re-treatment, and with another statement declaring no increased risk of nephrotoxicity or hematological toxicity (mainly thrombocytopenia) with RLT re-treatment. In the literature, very few patients experienced Grade 3-4 nephrotoxicity, while the reported rate of myelodysplastic syndrome or acute leukemia was 0%-2.2%.^80-91^

### Neoadjuvant Treatment

Table 4 depicts a summary of the role of neoadjuvant therapy in NET patients.

**Table 4.** Neoadyuvant therapies in neuroendocrine tumors.

Tumor characteristics				Most appropriated treatment
Locally advanced pancreatic NET nonresectable due to vascular invasion	Ki-67 ≤10%	SSTR expression		Chemotherapy to achieve resectability RLT to achieve resectability
			Progression to first-line chemotherapy that sought resectability	SSAs upon progression if resectability is not considered RLT to achieve resectability
			FDG-PET positive (SUVm 5-7)	Chemotherapy to achieve resectability
Pancreatic NETs	Ki-67 ≤10%	SSTR expression	Resectable ‘borderline’ liver metastases	Locoregional therapy followed by surgery RLT followed by surgery if response Chemotherapy followed by surgery if response
Intestinal NETs	Ki-67 ≤10%	SSTR expression	Locally advanced, nonresectable due to vascular invasion	SSAs upon progression if resectability is not considered RLT to achieve resectability
			Resectable ‘borderline’ liver metastases	Locoregional therapy followed by surgery RLT followed by surgery if response
Colorectal NETs	Ki-67 ≤10%	SSTR expression	Resectable ‘borderline’ liver metastases	Locoregional therapy followed by surgery
Bronchial NETs	G1-G2	SSTR expression	Large-volume, localized, and probable pneumonectomy	Surgery

Abbreviations: NETs, neuroendocrine tumors; G, grade; SSTR, somatostatin receptor; RLT, radioligand therapy; FDG-PET, 2-fluoro-2-deoxy-d-glucose-positron-electron tomography; SSAs, somatostatin analogs; SUV, standardized uptake value.

In locally advanced pancreatic NETs, which are considered nonresectable due to vascular invasion, with Ki-67 ≤10% and SSTR expression, chemotherapy (consensus, 85%) and RLT (majority, 65%) were considered adequate to attain resectability. Chemotherapy was deemed indicated in patients with a possible chance of achieving a response, thereby enabling surgery.^63^ In pancreatic NETs, there was not a clear Ki-67 cut-off value found for recommending chemotherapy, but generally speaking, a Ki-67 cut-off value between 5% and 20% was considered acceptable.^11^ In addition, published case studies involving pancreatic NET patients for whom RLT was prescribed reported promising results obtained with neoadjuvant treatment administered for subsequent surgery.^92,93^

When no response was obtained with chemotherapy in seeking resectability, the preferred options for second-line treatment were SSAs upon progression if resectability was not targeted (consensus, 80%) or RLT to achieve resectability (majority, 66%). According to published reports, RLT may play a role as neoadjuvant therapy in these patients.^94^

On the contrary, in locally advanced pancreatic NETs, which are considered unresectable due to vascular invasion, exhibiting Ki-67 ≤10%, SSTR expression, and fluorodeoxyglucose (FDG)-PET positivity (SUVm 5-7) as well, chemotherapy would seem adequate to target resectability (consensus, 88%). FDG-PET increased activity may indicate rapid progression of pancreatic NETs, even if these tumors are diagnosed at an early stage or turn out to be well-differentiated. This technique may enable an early identification of undifferentiated clones that affect the patient’s prognosis and outcome.^95^ In more aggressive or poorly differentiated tumors, chemotherapy appears an appropriate option.^11,63^

Considering pancreatic NETs, with Ki-67 ≤10%, SSTR expression, and resectable “borderline” liver metastases, experts considered the following appropriate: (a) loco-regional therapy (eg, yttrium-90) followed by surgery (unanimity, 100%); (b) RLT followed by surgery if response (consensus, 81%); (c) chemotherapy followed by surgery if response (consensus, 77%). Loco-regional treatments in advanced NETs (including pancreatic NETs) may induce cytotoxic and ischemic damage to metastases, thereby increasing the possibility of surgery.^12^ Regional control of liver metastases may be achieved (50%-80% 5-year OS in small retrospective series) with loco-regional therapy.^11,49,50,96-100^ These therapies may be considered as an alternative to systemic therapies if surgery of the primary tumor is envisioned, if the metastatic disease is limited to the liver. Concerning RLT, case series of advanced and (initially) unresectable pancreatic NETs have been published in which neoadjuvant RLT was proven successful, thereby enabling subsequent pancreatic surgery.^92,101,102^ The efficacy of chemotherapy as neoadjuvant treatment in advanced pancreatic NETs has been highlighted in observational studies.^11,62,63^

In locally advanced intestinal NETs, which are deemed unresectable due to vascular invasion, exhibiting Ki-67 ≤10% and SSTR expression, the Delphi participants considered either SSAs to be suitable upon progression if resectability was not considered (96% consensus) or RLT to achieve resectability (81% consensus). A few published cases were retrieved reporting that resectability of the primary tumor was achieved after administrating RLT as neoadjuvant therapy.^94^

Loco-regional therapy followed by surgery (96% consensus) and RLT followed by surgery in the event of response (81% consensus) were also considered appropriate in intestinal NETs, with Ki-67 ≤10%, SSTR expression, and resectable “borderline” liver metastases. As mentioned before, loco-regional therapy may prove effective in resectable borderline liver metastases of different NET types.^49,50,96,98-100,103^ Considering more specifically colorectal NETs, with Ki-67 ≤10%, SSTR expression, and resectable “borderline” liver metastases, loco-regional therapy followed by surgery would be appropriate (96% consensus). Loco-regional therapies may also play a role in colorectal NETs.^49,50,96,98-100^ The evidence of neoadjuvant RLT in intestinal NETs is still lacking.

Finally, in large-volume, localized, G1-G2 bronchial NETs with SSTR expression and a probable indication for pneumonectomy, the best strategy according to the experts consisted in primary tumor surgery (consensus, 77%). Currently, surgery is the most recommended option in these cases whenever possible.^13,18^

## Discussion

In the current project, we have identified different issues and controversies pertaining to NETs, critically evaluated the available evidence, and provided oncologists with specific information in the form of several statements that all have undergone a Delphi process. We have put a special focus on advanced cases and on the role of RLT.

First, we would like to highlight some general points. For the experts, given the variety of treatment options, heterogeneity of NETs, and individual disease complexities, it appeared strongly recommended that the most appropriate therapeutic strategy should be discussed within a NET-dedicated multidisciplinary team. As regard patient monitoring, oncologists should be aware that a combined approach consisting of clinical symptom evaluation, anatomical imaging, molecular imaging, and biomarker analysis is necessary to properly assess disease progression and treatment response, given that each of these factors presents with several limitations.^24,26-41^

Concerning advanced small intestine NETs, and in line with clinical guidelines, the efficacy and safety profile of RLT was considered superior to those of everolimus, and therefore recommended (if available) to be administered prior to the targeted drug in many cases.^39^ However, the proper treatment sequence needs to be further investigated, and it is currently evaluated in phase III RCTs. In this type of NETs with disease progression under SSAs and peritoneal carcinomatosis, RLT was also the preferred option. A retrospective study revealed that RLT may lead to bowel obstruction in patients with mesenteric or peritoneal disease. In a group of 81 patients with mesenteric or peritoneal disease, 6% experienced at least one bowel obstruction episode within a 3-month RLT therapy.^104^ However, the retrospective design and absence of control group makes it challenging to conclude whether RLT contributed to that or it was a consequence of the disease itself (the patients would have developed obstruction anyways). Therefore, certain centers prescribe short courses of prophylactic steroids, starting immediately after each dose of ^177^Lu-DOTATATE.^79^ Yet, further research is required to clarify this issue. Similarly, RLP was recommended in advanced gastrointestinal NETs with bone or liver metastases.

The experts similarly agreed on using SSAs as standard first-line therapy in functioning pancreatic advanced NETs. In the CLARINET study, lanreotide was associated with significantly prolonged PFS among patients with metastatic enteropancreatic NETs G1-2, with Ki-67 <10%.^53^ When advanced pancreatic NETs were analyzed, for asymptomatic patients with low tumor burden and disease progression under SSAs, everolimus or sunitinib were the preferred second-line treatment options.^40,54^ Given this scenario, achieving an objective response does not appear to be a treatment goal priority. Therefore, RLT and chemotherapy may not be given less preference over novel targeted therapies.^39,74,105-107^ Besides, oral treatment is more comfortable for patients. On the other hand, in case of functional NETs with early progression under SSAs, RLT was recommended.^43,55,56^ Finally, in high tumor burden cases, chemotherapy was the preferred option for the experts, as suggested in clinical guidelines.^11,18,39,61-64^

Next, different PPGL profiles were discussed. Consensus was obtained on recommending MIBG and ^177^Lu-DOTATATE in metastatic and progressing positive metaiodobenzylguanidine (MIBG) and SSTR expression PPGL, as well as SSAs in the event of functional, metastatic, and progressing PPGL cases. The evidence in these cases mainly comes from observational experiences,^65-68^ whereas the evidence for SAAs is still very scarce.^108^ Considering the effect of SSAs in functional NETs and efficacy of RLT in these patients, SAA may indeed be, at least hypothetically, a therapeutic reality. However, there was no consensus achieved for patients with metastatic and progressing PPGL with uptake on both FDG-PET-CT and SSTR scintigraphy. The therapeutic strategy for metastatic PPGL primarily aims to control excessive catecholamine secretion and tumor burden, given that are no curative treatment options available. The ESMO-EURACAN clinical guidelines for pheochromocytomas were published in 2020. For patients with metastatic PPGL, these guidelines recommended an individualized management approach in case of disease progression, including RLT, chemotherapy, local therapies, or additional treatments.^16^

In advanced pulmonary NETs G1-G2 with positive SSTR, the panel considered SSA to be the first-line treatment option.^69^ In disease progression cases under SAA, everolimus (consensus) and ^177^Lu-DOTATATE (majority) were the preferred options for second-line treatment.^53,70-72,74-77^ In a retrospective study, everolimus was found to be a valid therapeutic option for advanced, progressive, well-differentiated NETs, even in patients with positive FDG-PET.^73^ Therefore, as expressed by the experts, everolimus could even be considered in positive FDG-PET cases. In this patient subgroup, chemotherapy (TEMCAP) was also deemed a preferred therapy. Although without any robust evidence, it is likely that the association of FDG-PET with more aggressive tumors and a higher proliferative index influenced this decision.^109^

Data from observational studies have revealed that RLT retreatment can be a therapeutic choice for patients with progressive NETs.^80-91^ Therefore, there was a high agreement among experts concerning this therapeutic strategy. Moreover, as the maximum cumulative dose of ^177^Lu-DOTATATE has not yet been determined, the experts suggested that RLT retreatment could turn out to be preferred over targeted agents in pancreatic NETs for long responders. In fact, RLT retreatment was associated with similar or slightly longer PFS when indirectly compared with the RADIANT-4 results.^91^ In general, patients who showed reasonable response were those who retreated if the time to progression (TTP) was at least 1 year after completion of the last cycle of initial treatment or presented PFS ≥18 months from the first administration of initial RLT.^80-88,90,91^ Thus, the experts agreed on establishing a TTP of 1 year to consider retreatment. Similarly, retreatment with RLT was considered in well or moderately differentiated NETs with Ki67 proliferation index of 10%-20%, probably because of the impact of the Ki67 on response to RLT.^91^ Nevertheless, promising results have also been reported in patients who were retreated with a TTP <1 year or with Ki67 index >20%.^80^ However, in this section, there might have been some confusion/misunderstanding regarding safety issues with retreatment. The experts showed some concerns about the risk of increased hematological and kidney toxicity. The evidence indicates so far that common undesirable effects appear to be similar to those encountered during initial RLT. This unresolved issue may be due to the lack of well-designed studies that would definitively clarify these safety questions encountered with RLT retreatment.

Finally, the role of neoadjuvant therapies was discussed. In locally advanced NETs, in which surgery of the primary tumor is not considered, experts agreed on considering neoadjuvant therapies to seek resectability, such as chemotherapy and RLT in pancreatic NETs,^1-4^ or RLT in small intestine NETs.^42,43^ This treatment decision is fundamentally based on treatment response, but on other tumor characteristics, as well, including uptake in PET-FDG in pancreatic NETs, which would indicate chemotherapy as the preferred option.^11,63,95^ In patients with borderline or potentially resectable metastases, and despite the lack of robust evidence,^11,12,49,50,92,96-102^ a consensus was achieved to assess neoadjuvant treatment in selected patients, including loco-regional therapy (for pancreatic and enteric NETs), RLT (pancreatic and small intestine NETs), and even chemotherapy (pancreatic NETs) followed by surgery in case of response. On the other hand, there was consensus to consider surgery in pulmonary NETs, G1-G2 patients, with a probable indication for pneumonectomy, as recommended by clinical practice guidelines.^13,18^

## Conclusion

In summary, there are still many gaps regarding the management of patients with advanced NETs. The aim of this position document was to provide a guide in the decision-making process concerning NETs-affected patients, primarily focusing on those areas that might generate clinical questions or controversies in daily practice. For these cases, the experts’ recommendations through a Delphi process have proven to be a valid and useful tool. We believe that the practical framework provided in this document should be instrumental in helping health professionals better manage NET patients, while using RLT in these patients, as well.

## Supplementary Material

oyab041_suppl_supplementary_eTables

## Data Availability

The data underlying this article will be shared at reasonable request to the corresponding author.
