# Supplementary material for: Position Statement on the Diagnosis, Treatment, and Response Evaluation to Systemic Therapies of Advanced Neuroendocrine Tumors, With a Special Focus on Radioligand Therapy
Source: Oncologist. 2022 Mar 7;27(4):e328–39. doi: 10.1093/oncolo/oyab041 (PMC8982404; doi:10.1093/oncolo/oyab041)
Supplement: oyab041_suppl_supplementary_eTables [file oyab041_suppl_supplementary_etables.pdf]

The Oncologist
The official journal of the Society for Translational Oncology

Supplemental Tables for:
Position statement on the diagnosis, treatment, and response evaluation to systemic therapies of advanced neuroendocrine tumors
Jaume Capdevila et al.

Tables outline the results of the two Delphi processes pertaining to the whole project according to the proposed sections

Table S1. Overarching principles

#	Statement	Delphi round	Range of response (n=26)*				% of panel†	Final consensus‡
			4 (%)	3 (%)	2 (%)	1 (%)		
1	Newly diagnosed patients with NETs should be presented and discussed with a multidisciplinary NETs committee	1	0%	3%	35%	62%	97%	Unanimous (agreement)
		2	0%	0%	38%	62%	100%	
2	Improved nuclear medicine diagnostic techniques (especially ^{68}Ga--PET-CT) have had a relevant impact on treatment decision-making in patients with NETs	1	8%	8%	38%	46%	84%	Consensus (agreement)
		2	4%	8%	42%	46%	88%	
3	^{177}Lu-DOTATATE has restructured treatment schemes in patients with gastrointestinal NETs	1	0%	0%	15%	85%	100%	Unanimous (agreement)
		2	-	-	-	-	-	
4	^{177}Lu-DOTATATE has restructured treatment schemes in patients with pancreatic NETs	1	4%	15%	54%	27%	81%	Consensus (agreement)
		2	0%	15%	62%	23%	85%	

Abbreviations: NETs=neuroendocrine tumors; Ga-PET-CT=Gallium - positron electron tomography - computed tomography

*Delphi 1st and 2nd round response categories: 1=totally disagree; 2=basically disagree; 3=basically agree; 4=totally agree

†Rate of respondents who voted agree (1 or 2) or disagree (3 or 4) after each Delphi round

‡Final consensus was defined as: "unanimous" when the response rate in categories 1 and 2 or 3 or 4 was 100%; "consensus" when the response rate in categories 1 and 2 or 3 and 4 was 75-99%; "majority" when the response rate in categories 1 and 2 or 3 and 4 was 60%-74%; "dissent" if the response rate was <60%

Table S2. Progression and treatment response criteria in neuroendocrine tumors

#	Statement	Delphi round	Range of responses (n=26)*				% of panel†	Final consensus‡
			4 (%)	3 (%)	2 (%)	1 (%)		
5	Significant elevation in biological markers (*e.g.*, chromogranin A) translate disease progression	1	69%	31%	0%	0%	100%	Unanimous (disagreement)
		2	88%	12%	0%	0%	100%	
6	A recurrence or worsening of functional symptoms due to hormone production or new-onset symptoms determine disease progression	1	8%	42%	31%	19%	50%	Majority (disagreement)
		2	0%	73%	19%	8%	73%	
7	RECIST v 1.1 criteria determine lack of response to systemic therapies	1	4%	46%	31%	19%	50%	Consensus (disagreement)
		2	0%	81%	19%	0%	81%	
8	A significant increase in SUV in PET or an increased uptake using radiolabeled SSA (molecular imaging) determines disease progression	1	19%	58%	15%	8%	77%	Consensus (disagreement)
		2	8%	85%	8%	0%	92%	
9	CHOI criteria can only be applied in patients with anti-angiogenic treatment	1	8%	38%	27%	27%	54%	Dissent
		2	0%	58%	27%	15%	58%	

Abbreviations: PET=positron electron tomography; SUV=standardized uptake value; SSA=somatostatin analogue

*Delphi 1st and 2nd round response categories: 1=totally agree; 2=basically agree; 3=basically disagree; 4=totally disagree

†Rate of respondents who agreed (1 or 2) or disagreed (3 or 4) following each Delphi round

‡Final consensus was defined as: "unanimous" when the response rate in Categories 1 and 2 or Categories 3 or 4 was 100%; "consensus" when the response rate in Categories 1 and 2 or Categories 3 and 4 was 75-99%; "majority" when the response rate in Categories 1 and 2 or Categories 3 and 4 was 60%-74%; "dissent" if the response rate was <60%

Table S3. Treatment sequencing in gastrointestinal NETs

#	Statement	Delphi round	Range of response (n=26)*				% of panel†	Final consensus‡
			4 (%)	3 (%)	2 (%)	1 (%)		
10	In G1-G2 small intestine NETs, the efficacy and safety of RLT are superior to those of everolimus	1	8%	15%	58%	19%	77%	Consensus (agreement)
		2	0%	12%	85%	4%	89%	
11	In G1-G2 intestinal NETs others than small intestinal NETs, the efficacy and safety of RLT are superior to those of everolimus	1	19%	46%	23%	12%	65%	Consensus (disagreement)
		2	4%	88%	8%	0%	92%	
	In G2 small intestine NET with peritoneal carcinomatosis-only metastases, and in progression under SSAs, the...							
12	RLT is the most appropriate treatment	1	12%	31%	35%	23%	58%	Majority (agreement)
		2	0%	27%	62%	12%	74%	
13	Everolimus is the most appropriate treatment	1	12%	50%	23%	15%	62%	Consensus (disagreement)
		2	0%	85%	12%	4%	85%	
14	TEMCAP is the most appropriate treatment	1	35%	46%	15%	4%	81%	Consensus (disagreement)
		2	35%	62%	3%	0%	97%	
	In G2 gastrointestinal NETs with multiple bone metastases and progression under SSAs...							
15	RLT is the most appropriate treatment	1	4%	23%	42%	31%	73%	Consensus (agreement)
		2	0%	15%	73%	12%	85%	
16	Everolimus is the most appropriate treatment	1	8%	42%	42%	8%	50%	Majority (disagreement)
		2	0%	62%	38%	0%	62%	
17	TEMCAP is the most appropriate treatment	1	38%	31%	31%	0%	69%	Consensus (disagreement)
		2	62%	30%	8%	0%	92%	
	In patients with non-functional gastrointestinal NETs progressing under long-acting SSAs in whom RLT is considered, the most aropriate option is to...							
18	Stop definitively SSAs treatment	1	35%	42%	19%	4%	77%	Consensus (disagreement)
		2	15%	73%	8%	4%	88%	

19	Stop SSAs 4 weeks before RLT, and restart SSAs and use them between RLT cycles upon progression	1	15%	15%	50%	19%	69%	Consensus (agreement)
		2	4%	15%	73%	8%	81%	
20	Stop SSAs during RLT treatment and response or disease stabilization, and restart SSAs if progression	1	42%	38%	15%	5%	81%	Consensus (disagreement)
		2	62%	30%	8%	0%	82%	
	In functional gastrointestinal NETs with uncontrolled symptomatology progressing under long-acting SSA in whom treatment with RLT is considered...							
21	Long-acting SSAs will not be stopped and be continued until the start of RLT	1	31%	35%	15%	19%	66%	Majority (disagreement)
		2	27%	46%	19%	8%	73%	
22	Long-acting SSAs should be stopped and short term SSAs be started up to 24 hours before	1	12%	23%	27%	38%	65%	Consensus (agreement)
		2	0%	15%	27%	58%	85%	
23	Long-acting SSA should be stopped and other medical treatments (codeine, loperamide, cyproheptadine, etc.) be used to control symptoms	1	65%	23%	4%	8%	88%	Unanimous (disagreement)
		2	92%	8%	0%	0%	100%	
	In G1-G2 functional gastrointestinal NETs with liver metastases progressing under SSAs with uncontrolled hormonal symptoms, the most appropriate treatment is...							
24	RLT	1	0%	4%	38%	58%	96%	Consensus (agreement)
		2	0%	4%	31%	65%	96%	
25	Everolimus	1	15%	46%	31%	8%	61%	Consensus (disagreement)
		2	0%	85%	15%	0%	85%	
26	Loco-regional therapy	1	8%	30%	27%	35%	62%	Majority (agreement)
		2	4%	23%	27%	46%	73%	
27	Interferon	1	61%	35%	4%	0%	96%	Unanimous (disagreement)
		2	88%	12%	0%	0%	100%	
28	Shorten intervals or increase SSAs doses	1	19%	31%	31%	19%	50%	Dissent
		2	12%	42%	35%	12%	54%	
29	The short/long-term toxicity of RLT therapy is lower if RLT is given	1	23%	38%	27%	12%	61%	Consensus

	upon progression under SSA than if to other treatments such as everolimus	2	XX%	73%	12%	0%	88%	(disagreement)

Abbreviations: NETs=neuroendocrine tumors; G=grade; RLT=radiolingand therapy; SSAs=somatostatin analogues; TEMCAP=temozolamide + capecitabine.

*Delphi first and second round response categories: 1=totally agree; 2=basically agree; 3=basically disagree; 4=totally disagree

†Rate of respondents who voted agree (1 or 2) or disagreed (3 or 4) following each Delphi round

‡Final consensus was defined as: "unanimous" when the response rates in Categories 1 and 2 or Categories 3 or 4 was 100%; "consensus" when the response rates in Categories 1 and 2 or Categories 3 and 4 was 75-99%; "majority" when the response rate in Categories 1 and 2 or Categories 3 and 4 was 60%-74%; "dissent" if the response rate was <60%

Table S4. Sequencing of sequencing in pancreatic NETs

#	Statement	Delphi round	Range of responses (n=26)*				% of panel†	Final consensus‡
			4 (%)	3 (%)	2 (%)	1 (%)		
30	If there is no contraindication, systemic treatment with a SSA as single agent should be started in G1-G2 pancreatic NET patients	1	4%	23%	38%	35%	73%	Consensus (agreement)
		2	0%	19%	46%	35%	81%	
31	In asymptomatic patients with advanced pancreatic G1 NETs, low tumor burden, positive somatostatin receptor scintigraphy, in whom radiological progression is documented after more than 3 years of SSA treatment, a targeted therapy (everolimus and sunitinib) as second-line of treatment would be the preferred option	1	4%	15%	46%	35%	81%	Consensus (agreement)
		2	0%	12%	73%	15%	88%	
32	In functional, advanced pancreatic G1 NETs, positive somatostatin receptor scintigraphy, in which radiological progression is documented within the first 3 years of SSA treatment, second-line RLT would be the preferred option	1	8%	23%	38%	31%	69%	Consensus (agreement)
		2	0%	8%	84%	8%	92%	
33	In case of metastatic insulinoma in progression or with symptoms not well controlled under SSAs, everolimus would be preferred to RLT	1	3%	35%	35%	27%	62%	Consensus (agreement)
		2	0%	19%	73%	8%	81%	
34	It is recommended that patients with a Grade 1 or 2 pancreatic NET with somatostatin receptor expression receive at least one somatostatin analogue and targeted therapy or chemotherapy before radionuclide treatment is considered.	1	23%	23%	38%	15%	53%	Majority (agreement)
		2	12%	27%	58%	4%	62%	
35	In non-functional advanced G2 pancreatic NETs with symptoms resulting from high tumor burden, chemotherapy would be preferred over RLT	1	0%	15%	27%	58%	85%	Consensus (agreement)
		2	0%	4%	27%	69%	96%	

Abbreviations: NETs=neuroendocrine tumors; G=grade; RLT=radiolingand therapy; SSAs=somatostatin analogues

*Delphi 1st and 2nd rounds response categories: 1=totally agree; 2=basically agree; 3=basically disagree; 4=totally disagree

†Rate of respondents who voted agree (1 or 2) or disagree (3 or 4) following each Delphi round

‡Final consensus was defined as: "unanimous" when the response rate in Categories 1 and 2 or Categories 3 or 4 was 100%; "consensus" when the response rate in Categories 1 and 2 or Categories 3 and 4 was 75-99%; "majority" when the response rate in Categories 1 and 2 or Categories 3 and 4 was 60%-74%; "dissent" if the response rate was <60%

Table S5. Treatment sequencing in other NETs

#	Statement	Delphi round	Range of responses (n=26)*				% of panel†	Final consensus‡
			4 (%)	3 (%)	2 (%)	1 (%)		
36	In advanced pheochromocytomas / paragangliomas, Lu-DOTATATE is an accepted treatment in NETs with somatostatin receptor expression	1	0%	12%	50%	38%	88%	Consensus (agreement)
		2	0%	4%	73%	23%	96%	
	In progressive non-functional advanced pheochromocytomas /paragangliomas with somatostatin receptor expression and MIBG positive, the most appropriate treatment is...							
37	MIBG	1	0%	19%	46%	35%	81%	Consensus (agreement)
		2	0%	8%	73%	19%	92%	
38	^{177}Lu-DOTATATE	1	4%	31%	38%	27%	65%	Consensus (agreement)
		2	0%	23%	65%	12%	77%	
39	SSAs	1	19%	54%	27%	0%	73%	Consensus (disagreement)
		2	15%	77%	4%	4%	92%	
40	Chemotherapy; CVD	1	35%	38%	15%	12%	73%	Consensus (disagreement)
		2	31%	58%	12%	0%	88%	
41	Chemotherapy; temozolomide	1	35%	58%	0%	8%	92%	Unanimous (disagreement)
		2	31%	69%	0%	0%	100%	
	In advanced progressing functional pheochromocytomas / paragangliomas, with uncontrolled hormonal symptoms, somatostatin receptor expression and MIBG positive, the most appropriate treatment is...							
42	MIBG.	1	8%	19%	42%	31%	73%	Consensus (agreement)
		2	0%	8%	81%	12%	92%	
43	^{177}Lu-DOTATATE	1	4%	23%	42%	31%	73%	Consensus (agreement)
		2	0%	15%	65%	20%	85%	

44	SSAs	1	19%	19%	50%	12%	62%	Consensus (agreement)
		2	12%	11%	77%	0%	77%	
45	Chemotherapy, CVD	1	27%	46%	15%	12%	73%	Consensus (disagreement)
		2	20%	65%	15%	0%	85%	
46	Chemotherapy, temozolomide	1	26%	58%	8%	8%	84%	Unanimous (disagreement)
		2	19%	81%	0%	0%	100%	
	In advanced progressing pheochromocytomas /paragangliomas with somatostatin receptor expression and FDG-PET-CT uptake, the most appropriate treatment is...							
47	^{177}Lu-DOTATATE	1	8%	46%	38%	8%	54%	Majority (disagreement)
		2	0%	62%	38%	0%	62%	
48	SSAs	1	38%	46%	15%	0%	85%	Consensus (disagreement)
		2	23%	69%	8%	0%	92%	
49	Chemotherapy, CVD	1	4%	38%	31%	27%	58%	Majority (disagreement)
		2	0%	62%	31%	8%	62%	
50	Chemotherapy, temozolomide	1	19%	35%	31%	15%	54%	Majority (disagreement)
		2	12%	62%	23%	4%	74%	
	In advanced G1-G2 bronchial NETs with somatostatin receptor expression, the most appropriate 1st line treatment is...							
51	^{177}Lu-DOTATATE	1	38%	50%	8%	4%	88%	Consensus (disagreement)
		2	31%	65%	4%	0%	96%	
52	Everolimus	1	12%	38%	27%	23%	50%	Majority (disagreement)
		2	12%	62%	15%	12%	74%	
53	SSAs	1	0%	4%	46%	50%	96%	Unanimous (agreement)
		2	0%	0%	35%	65%	100%	
	In advanced G1-G2 bronchial NETs NETs with somatostatin receptor expression, progressing to SSAs, the most appropriate 2nd line treatment is....							
54	^{177}Lu-DOTATATE	1	12%	23%	54%	12%	66%	Majority (agreement)
		2	8%	27%	65%	0%	65%	

55	Everolimus	1	0%	12%	27%	61%	88%	Consensus (agreement)
		2	0%	4%	19%	77%	96%	
	In advanced G1-G2 bronchial NETs with somatostatin receptor expression and FDG-PET-CT uptake, the preferred treatment is							
56	^{177}Lu-DOTATATE	1	12%	54%	35%	0%	66%	Consensus (disagreement)
		2	12%	73%	15%	0%	85%	
57	Everolimus	1	8%	30%	35%	27%	62%	Majority (agreement)
		2	4%	23%	65%	8%	73%	
58	Chemotherapy; cisplatin and etoposide	1	27%	42%	23%	8%	69%	Consensus (disagreement)
		2	19%	65%	12%	4%	84%	
59	Chemotherapy; temozolomide, and capecitabine	1	4%	19%	46%	31%	77%	Consensus (agreement)
		2	0%	4%	88%	8%	96%	
	In advanced G1-G2 bronchial NETs, decisive factors for considering using ^{177}Lu-DOTATATE are...							
60	NETs with ≤10 mitoses/10 HPF and/or Ki-67<10%	1	8%	42%	31%	19%	50%	Consensus (disagreement)
		2	0%	77%	23%	0%	77%	
61	SUV intensity in Gallium-PET	1	8%	12%	30%	50%	80%	Consensus (agreement)
		2	0%	7%	31%	62%	93%	
62	Absence of FDG-PET-CT uptake	1	12%	54%	15%	19%	66%	Consensus (disagreement)
		2	0%	80%	12%	8%	80%	
63	Absence of radiological progression	1	58%	38%	4%	0%	96%	Unanimous (disagreement)
		2	81%	19%	0%	0%	100%	
64	Progression to everolimus	1	19%	15%	42%	23%	65%	Consensus (agreement)
		2	4%	4%	84%	8%	92%	
65	Progression to SSAs	1	4%	12%	46%	38%	84%	Consensus (agreement)
		2	0%	8%	62%	30%	92%	

Abbreviations: NETs=neuroendocrine tumors; G=grade; RLT=radiolingand therapy; SSAs=somatostatin analogues; CVD=cyclophosphamide, vincristine and dacarbazine; HPF=high-power fields; MIBG=metaiodobenzylguanidine; FDG-PET-CT=2-fluoro-2-deoxy-D-glucose- positron electron tomography - computed tomography; SUV=standardized uptake value

*Delphi 1st and 2nd rounds response categories: 1=totally agree; 2=basically agree; 3=basically disagree; 4=totally disagree

†Rate of respondents who voted agree (1 or 2) or disagree (3 or 4) following each Delphi round

‡Final consensus was defined as: "unanimous" when the response rate in Categories 1 and 2 or Categories 3 or 4 was 100%; "consensus" when the response rate in Categories 1 and 2 or Categories 3 and 4 was 75-99%; "majority" when the response rate in Categories 1 and 2 or Categories 3 and 4 was 60%-74%; "dissent" if the response rate was <60%

Table S6. RLT Re-treatment

#	Statement	Delphi round	Range of responses (n=30)*				% of panel†	Final consensus‡
			4 (%)	3 (%)	2 (%)	1 (%)		
66	Better results are expected with RLT re-treatment after progression to the first course of RLT (usually 4 doses) without other systemic therapies between the two RLT treatment courses	1	8%	62%	30%	0%	70%	Consensus (disagreement)
		2	8%	73%	19%	0%	81%	
67	Patients who have responded to a first RLT course of RLT are expected to respond to successive RLT courses	1	0%	15%	65%	20%	85%	Consensus (agreement)
		2	4%	0%	92%	4%	96%	
68	There appears to be no increased incidence of myelodysplastic syndrome or acute leukaemia with RLT re-treatments	1	14%	42%	38%	4%	56%	Majority (disagreement)
		2	7%	62%	31%	0%	69%	
69	As the maximum cumulative dose of ^{177}Lu-DOTATATE has not been determined yet, RLT re-treatment could be a preferable option to targeted therapy in pancreatic NETs for long responders	1	19%	23%	46%	12%	58%	Consensus (agreement)
		2	4%	19%	77%	0%	77%	
	An appropriate time to progression to consider RLT re-treatment is...							
70	<6 months	1	92%	8%	0%	0%	100%	Unanimous (disagreement)
		2	-	-	-	-	-	
71	6-12 months	1	30%	62%	4%	4%	92%	Unanimous (disagreement)
		2	15%	85%	0%	0%	100%	
72	12-18 months	1	0%	4%	81%	15%	96%	Consensus (agreement)
		2	-	-	-	-	-	
73	>18 months	1	4%	0%	11%	85%	96%	Consensus (agreement)
		2	-	-	-	-	-	
	Selection criteria for RLT re-treatment could be...							
74	Time to progression	1	4%	0%	15%	81%	96%	Consensus (agreement)
		2	-	-	-	-	-	

75	Ki-67	1	4%	54%	35%	8%	58%	Majority (disagreement)
		2	0%	73%	27%	0%	73%	
76	Treatment line number	1	8%	46%	35%	11%	54%	Majority (disagreement)
		2	0%	69%	31%	0%	69%	
77	Previous objective response	1	0%	8%	30%	62%	92%	Consensus (agreement)
		2	0%	4%	8%	88%	96%	
78	Tumor burden	1	0%	27%	65%	8%	73%	Consensus (agreement)
		2	0%	12%	88%	0%	88%	
79	Primary tumor location	1	0%	38%	46%	15%	62%	Majority (agreement)
		2	0%	27%	73%	0%	73%	
80	Further treatment options	1	5%	15%	65%	15%	80%	Consensus (agreement)
		2	0%	12%	88%	0%	88%	
	RLT re-treatment appears to be an option in well or moderately differentiated NETs, mainly with...							
81	Ki-67 10-20%	1	0%	4%	27%	69%	96%	Unanimous (agreement)
		2	0%	0%	27%	73%	100%	
82	Ki-67 20-30%	1	12%	46%	38%	4%	58%	Majority (disagreement)
		2	12%	62%	27%	0%	74%	
83	Ki-67 30-55%	1	38%	42%	20%	0%	80%	Consensus (disagreement)
		2	38%	54%	8%	0%	92%	
84	Ki-67 >55%	1	81%	15%	4%	0%	96%	Consensus (disagreement)
		2	-	-	-	-	-	
85	RLT re-treatment does not increase the risk of nephrotoxicity or haematological toxicity (mainly thrombopenia)	1	27%	42%	19%	12%	69%	Consensus (disagreement)
		2	15%	81%	4%	0%	96%	

Abbreviations: NETs=neuroendocrine tumors; RLT=radiolingand therapy

*Delphi [1st] and [2nd] rounds response categories: 1=totally agree; 2=basically agree; 3=basically disagree; 4=totally disagree

†Rate of respondents who voted agree (1 or 2) or disagree (3 or 4) followoing each Delphi round

‡Final consensus was defined as: "unanimous" when the response rate in Categories 1 and 2 or Categories 3 or 4 was 100%; "consensus" when the response rate in Categories 1 and 2 or Categories 3 and 4 was 75-99%; "majority" when the response rate in Categories 1 and 2 or Categories 3 and 4 was 60%-74%; "dissent" if the response rate was <60%

Table S7. Neoadjuvant treatment

#	Statement	Delphi round	Range of responses (n=30)*				% of panel†	Final consensus‡
			4 (%)	3 (%)	2 (%)	1 (%)		
	In locally advanced pancreatic NETs, with somatostatin receptor expression, Ki67≤10%, unresectable due to vascular invasion, it would be considered appropriated...							
86	SSAs upon progression, without seeking resectability	1	23%	31%	23%	23%	54%	Majority (disagreement)
		2	12%	62%	18%	8%	74%	
87	Chemotherapy to achieve resectability	1	19%	8%	42%	31%	73%	Consensus (agreement)
		2	8%	8%	81%	4%	85%	
88	RLT to achieve resectability	1	23%	23%	46%	8%	54%	Majority (agreement)
		2	19%	15%	65%	0%	65%	
89	Sunitinib to achieve resectability	1	46%	35%	15%	4%	81%	Consensus (disagreement)
		2	62%	34%	0%	4%	96%	
90	Lenvatinib (off-label) to achieve resectability	1	58%	26%	12%	4%	84%	Consensus (disagreement)
		2	73%	19%	8%	0%	92%	
91	Everolimus to achieve resectability	1	65%	27%	4%	4%	92%	Unanimous (disagreement)
		2	85%	15%	0%	0%	100%	
	In locally advanced pancreatic NETs, with somatostatin receptor expression, Ki-67≤10%, unresectable due to vascular invasion, in which first-line chemotherapy that sought resectability did not suceed, an appropriated second-line treatment might be...							
92	SSAs under progression, without seeking resectability.	1	15%	23%	27%	35%	62%	Consensus (agreement)
		2	4%	16%	42%	38%	80%	
93	Sunitinib to achieve resectability	1	42%	35%	15%	8%	77%	Unanimous (disagreement)
		2	65%	35%	0%	0%	100%	
94	Lenvatinib (off-label) to achieve resectability	1	50%	19%	31%	0%	69%	Consensus

		2	70%	15%	15%	0%	85%	(disagreement)
95	RLT to achieve resectability	1	19%	19%	54%	8%	62%	Majority (agreement)
		2	12%	23%	62%	4%	66%	
96	Everolimus to achieve resectability	1	58%	30%	8%	4%	88%	Unanimous (disagreement)
		2	88%	12%	0%	0%	100%	
	In locally advanced pancreatic NETs, with somatostatin receptor expression, Ki67≤10%, unresectable due to vascular invasion, FDG-PET uptake (SUVm 5-7), it would be considered appropriated...							
97	SSA upon progression, without seeking resectability	1	27%	35%	27%	12%	62%	Consensus (disagreement)
		2	23%	58%	15%	4%	81%	
98	Chemotherapy to achieve resectability	1	0%	16%	38%	46%	84%	Consensus (agreement)
		2	0%	12%	35%	54%	88%	
99	RLT to achieve resectability	1	31%	42%	27%	0%	73%	Consensus (disagreement)
		2	19%	58%	23%	0%	77%	
100	Sunitinib to achieve resectability	1	50%	35%	12%	3%	85%	Unanimous (disagreement)
		2	69%	31%	0%	0%	100%	
101	Lenvatinib off-label to achieve resectability	1	50%	35%	15%	0%	85%	Consensus (disagreement)
		2	73%	19%	8%	0%	92%	
102	Everolimus to achieve resectability	1	58%	30%	8%	4%	88%	Unanimous (disagreement)
		2	81%	19%	0%	0%	100%	
	In locally advanced intestinal NETs, with somatostatin receptor expression, Ki-67≤10%, unresectable due to vascular invasion, it would be considered appropriated...							
103	SSA upon progression, without seeking resectability.	1	15%	19%	42%	23%	67%	Majority (agreement)
		2	8%	19%	65%	8%	73%	
104	RLT to achieve resectability	1	19%	12%	50%	19%	69%	Consensus (agreement)
		2	8%	8%	85%	0%	85%	
105	Everolimus to achieve resectability	1	65%	27%	4%	4%	92%	Unanimous (disagreement)
		2	85%	15%	0%	0%	100%	

106	Lenvatinib (off-label) to achieve resectability	1	58%	31%	11%	0%	89%	Unanimous (disagreement)
		2	81%	19%	0%	0%	100%	
107	Chemotherapy to achieve resectability	1	46%	42%	12%	0%	88%	Unanimous (disagreement)
		2	62%	38%	0%	0%	100%	
	In intestinal NETs, with somatostatin receptor expression, Ki-67≤10%, and resectable 'borderline' liver metastases, it would be considered appropriated...							
108	Surgery	1	15%	38%	19%	27%	53%	Majority (disagreement)
		2	12%	50%	19%	19%	62%	
109	Loco-regional therapy (*e.g.,* yttrium-90, TACE...) followed by surgery	1	0%	15%	38%	47%	85%	Consensus (agreement)
		2	0%	4%	54%	42%	96%	
110	Loco-regional therapy (e.g., yttrium-90, TACE...) without surgery	1	35%	46%	15%	4%	81%	Unanimous (disagreement)
		2	19%	81%	0%	0%	100%	
111	RLT followed by surgery if response	1	12%	18%	58%	12%	70%	Consensus (agreement)
		2	4%	15%	81%	0%	81%	
112	RLT without surgery	1	42%	50%	8%	0%	92%	Unanimous (disagreement)
		2	31%	69%	0%	0%	100%	
113	SSAs upon progression	1	38%	27%	31%	4%	65%	Consensus (disagreement)
		2	62%	19%	19%	0%	81%	
	In pancreatic NETs, with somatostatin receptor expression, Ki-67≤10%, and resectable 'borderline' liver metastases, it would be considered appropriate...							
114	Surgery	1	42%	23%	12%	23%	65%	Consensus (disagreement)
		2	54%	26%	8%	12%	80%	
115	Loco-regional therapy (*e.g.,* yttrium-90, TACE, etc.) followed by surgery	1	4%	19%	58%	19%	7%	Unanimous (agreement)
		2	0%	0%	88%	12%	100%	
116	Loco-regional therapy (e.g., yttrium-90, TACE, etc) without surgery	1	35%	38%	27%	0%	73%	Consensus (disagreement)
		2	38%	58%	4%	0%	96%	
117	RLT followed by surgery if response	1	23%	27%	42%	8%	50%	Consensus (agreement)
		2	8%	12%	81%	0%	81%	

118	RLT without surgery	1	50%	38%	12%	0%	88%	Unanimous (disagreement)
		2	65%	35%	0%	0%	100%	
119	SSAs upon progression	1	38%	35%	27%	0%	73%	Consensus (disagreement)
		2	50%	38%	12%	0%	88%	
120	Chemotherapy followed by surgery if response	1	15%	19%	38%	28%	66%	Consensus (agreement)
		2	12%	11%	65%	12%	77%	
	In colorectal NETs, with somatostatin receptor expression, Ki67≤10%, and resectable 'borderline' liver metastases. it would be considered appropriated...							
121	Surgery	1	27%	35%	15%	23%	62%	Majority (disagreement)
		2	31%	42%	12%	15%	73%	
122	Loco-regional therapy (e.g., yttrium-90, TACE, etc.) followed by surgery	1	0%	15%	50%	35%	85%	Consensus (agreement)
		2	0%	4%	81%	15%	96%	
123	Loco-regional therapy (e.g., yttrium-90, TACE, etc) without surgery.	1	27%	42%	27%	4%	69%	Unanimous (disagreement)
		2	19%	81%	0%	0%	100%	
124	RLT followed by surgery if response	1	19%	35%	35%	11%	54%	Dissent
		2	8%	38%	50%	4%	54%	
125	RLT without surgery	1	38%	54%	8%	0%	92%	Unanimous (disagreement)
		2	23%	77%	0%	0%	100%	
126	SSAs unpon progression	1	31%	42%	19%	8%	73%	Consensus (disagreement)
		2	27%	69%	4%	0%	96%	
127	Chemotherapy followed by surgery if response	1	38%	31%	31%	0%	69%	Consensus (disagreement)
		2	54%	27%	19%	0%	81%	
	In large, localised, G1-G2 bronchial NETs, with somatostatin receptor expression and a probable indication for pneumonectomy, the best treatment strategy is...							
128	Surgery	1	27%	4%	31%	38%	69%	Consensus (agreement)
		2	23%	0%	31%	46%	77%	
129	RLT followed by surgery if response	1	15%	50%	35%	0%	65%	Majority (disagreement)
		2	4%	62%	35%	0%	65%	

130	SSAs upon progression	1	46%	35%	15%	4%	81%	Consensus (disagreement)
		2	69%	23%	8%	0%	92%	
131	Everolimus followed by surgery	1	46%	38%	12%	4%	84%	Consensus (disagreement)
		2	54%	42%	4%	0%	96%	
132	Chemotherapy followed by surgery	1	15%	35%	38%	12%	50%	Dissent
		2	12%	31%	54%	4%	58%	

Abbreviations: NETs=neuroendocrine tumors; G=grade; RLT=radiolingand therapy; SSAs=somatostatin analogues; TACE=transarterial chemoembolization; FDG-PET=2-fluoro-2-deoxy-D-glucose- positron electron tomography; SUV=standardized uptake value

*Delphi [1st] and [2nd] rounds response categories: 1=totally agree; 2=basically agree; 3=basically disagree; 4=totally disagree

†Rate of respondents who voted agree (1 or 2) or disagree (3 or 4) after each Delphi round

‡Final consensus was defined as: "unanimous" when the response rate in Categories 1 and 2 or Categories 3 or 4 was 100%; "consensus" when the response rate in Categories 1 and 2 or Categories 3 and 4 was 75-99%; "majority" when the response rate in categories 1 and 2 or Categories 3 and 4 was 60%-74%; "dissent" if the response rate was <60%
